# Double-Framed Soft Hypervector Spaces

**DOI:** 10.1155/2014/451928

**Published:** 2014-04-03

**Authors:** G. Muhiuddin, Abdullah M. Al-Roqi

**Affiliations:** ^1^Department of Mathematics, University of Tabuk, Tabuk 71491, Saudi Arabia; ^2^Department of Mathematics, King Abdulaziz University, Jeddah 21589, Saudi Arabia

## Abstract

The notions of double-framed soft subfields, double-framed soft algebras over double-framed soft subfields, and double-framed soft hypervector spaces are introduced, and their properties and characterizations are considered.

## 1. Introduction

The concept of soft set has been introduced by Molodtsov in 1999 [1] as a new mathematical tool for dealing with uncertainties. Later on, some research papers have appeared on the algebraic structures of soft set theory dealing with uncertainties. In 2007, Aktaş and Ça$\overset{˘}{\text{g}}$man [2] studied the basic concepts of soft set theory and compared soft sets to fuzzy and rough sets, providing examples to clarify their differences. They also discussed the notion of soft groups. Ça$\overset{˘}{\text{g}}$man and Engino$\overset{˘}{\text{g}}$lu [3] introduced fuzzy parameterized (FP) soft sets and investigated their related properties. They proposed a decision making method based on FP-soft set theory and provided an example which shows that the method can be successfully applied to the problems that contain uncertainties. Feng [4] considered the application of soft rough approximations in multicriteria group decision making problems. Recently, many algebraic properties of soft sets are studied (see [5–10]).

The hyperstructure theory was introduced by Marty [11] at the 8th Congress of Scandinavian Mathematicians in 1934. As a generalization of fuzzy vector spaces, the fuzzy hypervector spaces are studied by Ameri and Dehghan (see [12, 13]).

In this paper, using the notion of DFS sets which is introduced in [14], we introduce the notions of double-framed soft subfields, double-framed soft algebras over double-framed soft subfields, and double-framed soft hypervector spaces, and then we investigate their properties.

## 2. Preliminaries

A map ∘:*H* × *H* → *P*_∗_(*H*) is called a hyperoperation or join operation, where *P*_∗_(*H*) is the set of all nonempty subsets of *H*. The join operation is extended to subsets of *H* in natural way, so that *A*∘*B* is given by

(1) $$\begin{array}{c} A\circ B=⋃\left\{a\circ b\;|\;a\in A,b\in B\right\}. \end{array}$$

The notations *a*∘*A* and *A*∘*a* are used for {*a*}∘*A* and *A*∘{*a*}, respectively. Generally, the singleton {*a*} is identified by its element *a*.

**Definition 1 (see [15])**

Let *F* be a field and (*V*, +) an abelian group. A* hypervector space* over *F* is defined to be the quadruplet (*V*, +, ∘, *F*), where “∘” is a mapping

(2) $$\begin{array}{c} \circ :F\times V⟶P_{*}\left(V\right), \end{array}$$

such that for all *a*, *b* ∈ *F* and *x*, *y* ∈ *V* the following conditions hold:

(H1) *a*∘(*x* + *y*)⊆*a*∘*x* + *a*∘*y*,
(H2) (*a* + *b*)∘*x*⊆*a*∘*x* + *b*∘*x*,
(H3) *a*∘(*b*∘*x*) = (*ab*)∘*x*,
(H4) *a*∘(−*x*) = (−*a*)∘*x* = −(*a*∘*x*),
(H5) *x* ∈ 1∘*x*.

A hypervector space (*V*, +, ∘, *F*) over a field *F* is said to be* strongly left distributive* (see [12]) if it satisfies the following condition:

(3) $$\begin{array}{c} \left(\forall a\in F\right)\;\;\left(\forall x,y\in V\right)\;\;\left(a\circ \left(x+y\right)=a\circ x+a\circ y\right). \end{array}$$

Molodtsov [1] defined the soft set in the following way. Let *U* be an initial universe set and let *E* be a set of parameters. We say that the pair (*U*, *E*) is a* soft universe.* Let *P*(*U*) denote the power set of *U* and *A*, *B*, *C*,…⊆*E*.

**Definition 2 (see [1])**

A pair $\left(\tilde{f},A\right)$ is called a* soft set* over *U*, where $\tilde{f}$ is a mapping given by

(4) $$\begin{array}{c} \tilde{f}:A⟶P\left(U\right). \end{array}$$

In other words, a soft set over *U* is a parameterized family of subsets of the universe *U*. For *ε* ∈ *A*, $\tilde{f}(\varepsilon )$ may be considered as the set of *ε*-approximate elements of the soft set $\left(\tilde{f},A\right)$. Clearly, a soft set is not a set. For illustration, Molodtsov considered several examples in [1].

Let *E* be a set of parameters, and *A*, *B*, *C*,… are subsets of *E*.

**Definition 3 (see [14])**

A double-framed pair $\langle (\tilde{f},\alpha );A\rangle$ is called a* double-framed soft set* (briefly,* DFS-set*) over *U* where $\tilde{f}$ and *α* are mappings from *A* to *P*(*U*).

For a DFS-set $\langle (\tilde{f},\alpha );A\rangle$ over *U* and two subsets *γ* and *δ* of *U*, the *γ-inclusive set* and the *δ-exclusive set* of $\langle (\tilde{f},\alpha );A\rangle$, denoted by $i_{A}(\tilde{f};\gamma )$ and *e*_*A*_(*α*; *δ*), respectively, are defined as follows:

(5) $$\begin{array}{c} i_{A}\left(\tilde{f};\gamma \right)≔\left\{x\in A\;|\;\gamma \subseteq \tilde{f}\left(x\right)\right\}, \end{array}$$

(6) $$\begin{array}{c} e_{A}\left(\alpha ;\delta \right)≔\left\{x\in A\;|\;\delta ⊇\alpha \left(x\right)\right\}, \end{array}$$

respectively. The set

(7) $$\begin{array}{c} DF_{A}{\left(\tilde{f},\alpha \right)}_{\left(\gamma ,\delta \right)}≔\left\{x\in A\;|\;\gamma \subseteq \tilde{f}\left(x\right),\delta ⊇\alpha \left(x\right)\right\} \end{array}$$

is called a* double-framed including set* of $\langle (\tilde{f},\alpha );A\rangle$. It is clear that

(8) $$\begin{array}{c} DF_{A}{\left(\tilde{f},\alpha \right)}_{\left(\gamma ,\delta \right)}=i_{A}\left(\tilde{f};\gamma \right)\cap e_{A}\left(\alpha ;\delta \right). \end{array}$$

## 3. Double-Framed Soft Algebras over Double-Framed Soft Subfields

In what follows, let *F* be a field unless otherwise specified.

**Definition 4**

A double-framed soft set $\langle (\tilde{f},\alpha );F\rangle$ over *U* is called a* double-framed soft subfield* of *F* if the following conditions are satisfied:

1. $\left(\forall a,b\in F\right)\;\;\left(\tilde{f}\left(a+b\right)⊇\tilde{f}\left(a\right)\cap \tilde{f}\left(b\right),\;\;\alpha \left(a+b\right)\subseteq \alpha \left(a\right)\cup \alpha \left(b\right)\right)$,
2. $\left(\forall a\in F\right)\;\;\left(\tilde{f}\left(-a\right)⊇\tilde{f}\left(a\right),\;\;\alpha \left(-a\right)\subseteq \alpha \left(a\right)\right)$,
3. $\left(\forall a,b\in F\right)\;\;\left(\tilde{f}\left(a\right)\cap \tilde{f}\left(b\right)\subseteq \tilde{f}\left(ab\right),\;\;\alpha \left(a\right)\cup \alpha \left(b\right)⊇\alpha \left(ab\right)\right)$,
4. $\left(\forall a\in F\right)\;\;\left(a\neq 0\Rightarrow \tilde{f}\left(a\right)\subseteq \tilde{f}\left(a^{-1}\right),\;\;\alpha \left(a\right)⊇\alpha \left(a^{-1}\right)\right)$.

**Proposition 5**

If $\langle (\tilde{f},\alpha );F\rangle$ is a double-framed soft subfield of *F*, then

1. $\left(\forall a\in F\right)\;\;\left(\tilde{f}\left(a\right)\subseteq \tilde{f}\left(0\right),\;\;\alpha \left(a\right)⊇\alpha \left(0\right)\right)$,
2. $\left(\forall a\in F\right)\;\;\left(a\neq 0\Rightarrow \tilde{f}\left(a\right)\subseteq \tilde{f}\left(1\right),\;\;\alpha \left(a\right)⊇\alpha \left(1\right)\right)$,
3. $\tilde{f}(1)\subseteq \tilde{f}(0)$ *and α*(1)⊇*α*(0).

**Proof**

(1) For all *a* ∈ *F*, we have

(9) $$\begin{array}{c} \tilde{f}\left(0\right)=\tilde{f}\left(a+\left(-a\right)\right)⊇\tilde{f}\left(a\right)\cap \tilde{f}\left(-a\right)=\tilde{f}\left(a\right),\\ \alpha \left(0\right)=\alpha \left(a+\left(-a\right)\right)\subseteq \tilde{f}\left(a\right)\cup \tilde{f}\left(-a\right)=\tilde{f}\left(a\right). \end{array}$$

(2) Let *a* ∈ *F* be such that *a* ≠ 0. Then

(10) $$\begin{array}{c} \tilde{f}\left(1\right)=\tilde{f}\left(aa^{-1}\right)⊇\tilde{f}\left(a\right)\cap \tilde{f}\left(a^{-1}\right)=\tilde{f}\left(a\right),\\ \alpha \left(1\right)=\alpha \left(aa^{-1}\right)\subseteq \alpha \left(a\right)\cup \alpha \left(a^{-1}\right)=\alpha \left(a\right). \end{array}$$

(3) It is by (1).

It is easy to show that the following theorem holds.

**Theorem 6**

A soft set $\langle (\tilde{f},\alpha );F\rangle$ over *U* is a double-framed soft subfield of *F* if and only if the nonempty *γ*-inclusive set

(11) $$\begin{array}{c} i_{F}\left(\tilde{f};\gamma \right)≔\left\{a\in F\;|\;\gamma \subseteq \tilde{f}\left(a\right)\right\} \end{array}$$

and the nonempty *δ*-exclusive set

(12) $$\begin{array}{c} e_{F}\left(\alpha ;\delta \right)≔\left\{a\in F\;|\;\delta ⊇\alpha \left(a\right)\right\}, \end{array}$$

of $\langle (\tilde{f},\alpha );F\rangle$, are subfields of *F* for all *γ*, *δ* ∈ *P*(*U*).

**Definition 7**

Let *V* be algebra over *F* and let $\langle (\tilde{f},\alpha );F\rangle$ be a double-framed soft subfield over *U*. A double-framed soft set $\langle (\tilde{g},\beta );V\rangle$ is called a* double-framed soft algebra* over $\langle (\tilde{f},\alpha );F\rangle$ if it satisfies the following conditions:

1. $\left(\forall x,y\in V\right)\;\;\left(\tilde{g}\left(x+y\right)⊇\tilde{g}\left(x\right)\cap \tilde{g}\left(y\right),\;\;\beta \left(x+y\right)\subseteq \beta \left(x\right)\cup \beta \left(y\right)\right)$,
2. $\left(\forall a\in F\right)\;\;\left(\forall x\in V\right)\;\;\left(\tilde{g}\left(ax\right)⊇\tilde{f}\left(a\right)\cap \tilde{g}\left(x\right),\;\;\beta \left(ax\right)\subseteq \alpha \left(a\right)\cup \beta \left(x\right)\right)$,
3. $\left(\forall x,y\in V\right)\;\;\left(\tilde{g}\left(xy\right)⊇\tilde{g}\left(x\right)\cap \tilde{g}\left(y\right),\;\;\beta \left(xy\right)\subseteq \beta \left(x\right)\cup \beta \left(y\right)\right)$,
4. $\left(\forall x\in V\right)\;\;\left(\tilde{f}\left(1\right)⊇\tilde{g}\left(x\right),\;\;\alpha \left(1\right)\subseteq \beta \left(x\right)\right)$.

**Proposition 8**

Let *V* be an algebra over *F* and let $\langle (\tilde{f},\alpha );F\rangle$ be a double-framed soft subfield over *U*. If $\langle (\tilde{g},\beta );V\rangle$ is a double-framed soft algebra over $\langle (\tilde{f},\alpha );F\rangle$, then $\tilde{f}(0)⊇\tilde{g}(x)$ and *α*(0)⊆*β*(*x*) for all *x* ∈ *V*.

**Proof**

For any *x* ∈ *V*, we have $\tilde{f}(0)⊇\tilde{f}(1)⊇\tilde{g}(x)$ and *α*(0)⊆*α*(1)⊆*β*(*x*).

**Theorem 9**

Let *V* be an algebra over *F* and let $\langle (\tilde{f},\alpha );F\rangle$ be a double-framed soft subfield over *U*. Then, a double-framed soft set $\langle (\tilde{g},\beta );V\rangle$ is a double-framed soft algebra over $\langle (\tilde{f},\alpha );F\rangle$  if and only if the following conditions are valid:

1. $(\forall a,b\in F)\;\;(\forall x,y\in V)\;\;\left(\begin{array}{c} \tilde{g}\left(ax+by\right)⊇\left(\tilde{f}\left(a\right)\cap \tilde{g}\left(x\right)\right)\cap \left(\tilde{f}\left(b\right)\cap \tilde{g}\left(y\right)\right),\\ \beta \left(ax+by\right)\subseteq \left(\alpha \left(a\right)\cup \beta \left(x\right)\right)\cup \left(\alpha \left(b\right)\cup \beta \left(y\right)\right) \end{array}\right)$,
2. $(\forall x,y\in V)\;\;\left(\tilde{g}(xy)⊇\tilde{g}(x)\cap \tilde{g}(y),\;\;\beta (xy)\subseteq \beta (x)\cup \beta (y)\right)$,
3. $(\forall x\in V)\;\;\left(\tilde{f}(1)⊇\tilde{g}(x),\;\;\alpha (1)\subseteq \beta (x)\right)$.

**Proof**

Assume that $\langle (\tilde{g},\beta );V\rangle$ is a double-framed soft algebra over $\langle (\tilde{f},\alpha );F\rangle$. By using (1) and (2) of Definition 7, we have

(13) g~(ax+by)⊇g~(ax)∩g~(by)⊇(f~(a)∩g~(x))∩(f~(b)∩g~(y)),β(ax+by)⊆β(ax)∪β(by)⊆(α(a)∪β(x))∪(α(b)∪β(y)),

for all *a*, *b* ∈ *F* and *x*, *y* ∈ *V*. Also conditions (2) and (3) are hold by Definition 7(3) and Definition 7(4), respectively.

Conversely, suppose that $\langle (\tilde{g},\beta );V\rangle$ satisfies three conditions (1), (2), and (3). Then,

(14) g~(x+y)=g~(1x+1y)⊇(f~(1)∩g~(x))∩(f~(1)∩g~(y))=g~(x)∩g~(y),

(15) β(x+y)=β(1x+1y)⊆(α(1)∪β(x))∪(α(1)∪β(y))=β(x)∪β(y).

The condition (3) and Proposition 5(3) imply that $\tilde{f}(0)⊇\tilde{f}(1)⊇\tilde{g}(x)$ and *α*(0)⊆*α*(1)⊆*β*(*x*) for all *x* ∈ *V*. Thus,

(16) g~(ax)=g~(ax+0x)⊇(f~(a)∩g~(x))∩(f~(0)∩g~(x))=(f~(a)∩g~(x))∩g~(x)=f~(a)∩g~(x),

(17) β(ax)=β(ax+0x)⊆(α(a)∪β(x))∪(α(0)∪β(x))=(α(a)∪β(x))∪β(x)=α(a)∪β(x),

for all *a* ∈ *F* and *x* ∈ *V*. Therefore, $\langle (\tilde{g},\beta );V\rangle$ is a double-framed soft algebra over $\langle (\tilde{f},\alpha );F\rangle$.

## 4. Double-Framed Soft Hypervector Spaces

**Definition 10**

Let *V* be a hypervector space over *F* and $\langle (\tilde{f},\alpha );F\rangle$ a double-framed soft subfield of *F*. A soft set $\langle (\tilde{g},\beta );V\rangle$ over *V* is called a* double-framed soft hypervector space* of *V* related to $\langle (\tilde{f},\alpha );F\rangle$ if the following assertions are valid:

1. $\left(\forall x,y\in V\right)\;\;\left(\tilde{g}\left(x+y\right)⊇\tilde{g}\left(x\right)\cap \tilde{g}\left(y\right),\;\;\beta \left(x+y\right)\subseteq \beta \left(x\right)\cup \beta \left(y\right)\right)$,
2. $\left(\forall x\in V\right)\;\;\left(\tilde{g}\left(-x\right)⊇\tilde{g}\left(x\right),\;\;\beta \left(-x\right)\subseteq \beta \left(x\right)\right)$,
3. $\left(\forall a\in F\right)\;\;\left(\forall x\in V\right)\;\;\left({⋂}_{y\in a\circ x}\tilde{g}\left(y\right)⊇\tilde{f}\left(a\right)\cap \tilde{g}\left(x\right),\;\;{⋃}_{y\in a\circ x}\beta \left(y\right)\subseteq \alpha \left(a\right)\cup \beta \left(x\right)\right)$,
4. $\tilde{f}(1)⊇\tilde{g}(\theta )$ and *α*(1)⊆*β*(*θ*) where *θ* is the zero of (*V*, +).

**Proposition 11**

Let *V* be a hypervector space over *F* and $\langle (\tilde{f},\alpha );F\rangle$ a double-framed soft subfield of *F*. If $\langle (\tilde{g},\beta );V\rangle$ is a double-framed soft hypervector space of *V* related to $\langle (\tilde{f},\alpha );F\rangle$, then

1. $\tilde{g}(\theta )\subseteq \tilde{f}(0)$ *and β*(*θ*)⊇*α*(0),
2. $\left(\forall x\in V\right)\;\;\left(\tilde{g}\left(x\right)\subseteq \tilde{g}\left(\theta \right),\;\;\beta \left(x\right)⊇\beta \left(\theta \right)\right)$,
3. $\left(\forall x\in V\right)\;\;\left(\tilde{g}\left(x\right)\subseteq \tilde{f}\left(0\right),\;\;\beta \left(x\right)⊇\alpha \left(0\right)\right)$.

**Proof**

It is an immediate consequence of Definition 10 and Proposition 5.

**Proposition 12**

Let *V* be a hypervector space over *F*. If $\langle (\tilde{g},\beta );V\rangle$ is a double-framed soft hypervector space of *V* related to a double-framed soft subfield $\langle (\tilde{f},\alpha );F\rangle$ of *F*, then

(18) $$\begin{array}{c} \left(\forall x\in V\right)\;\;\left(\tilde{g}\left(x\right)=\underset{y\in 1\circ x}{⋂}\tilde{g}\left(y\right),\beta \left(x\right)=\underset{y\in 1\circ x}{⋃}\beta \left(y\right)\right). \end{array}$$

**Proof**

Let *x* ∈ *V*. Since *x* ∈ 1∘*x* by (H5), we have $\tilde{g}(x)⊇{⋂}_{y\in 1\circ x}\tilde{g}(y)$ and *β*(*x*)⊆⋂_*y*∈1∘*x*_ *β*(*y*).

By using Definition 10(3) and

(19) $$\begin{array}{c} \underset{y\in 1\circ x}{⋂}\tilde{g}\left(y\right)⊇\tilde{f}\left(1\right)\cap \tilde{g}\left(x\right)⊇\tilde{g}\left(x\right),\\ \underset{y\in 1\circ x}{⋃}\beta \left(y\right)\subseteq \alpha \left(1\right)\cup \beta \left(x\right)\subseteq \beta \left(x\right), \end{array}$$

we get $\tilde{g}(x)={⋂}_{y\in 1\circ x}\tilde{g}(y)$ and *β*(*x*) = ⋃_*y*∈1∘*x*_ *β*(*y*) for all *x* ∈ *V*.

**Theorem 13**

Assume that a hypervector space *V* over *F* is strongly left distributive. Let $\langle (\tilde{f},\alpha );F\rangle$ be a double-framed soft subfield of *F*. Then, a DFS-set $\langle (\tilde{g},\beta );V\rangle$ over *V* is a double-framed soft hypervector space of *V* related to $\langle (\tilde{f},\alpha );F\rangle$ if and only if the following conditions are true:

1. ${⋂}_{z\in a\circ x+b\circ y}\tilde{g}\left(z\right)⊇\left(\tilde{f}\left(a\right)\cap \tilde{g}\left(x\right)\right)\cap \left(\tilde{f}\left(b\right)\cap \tilde{g}\left(y\right)\right)$ *and *⋃_*z*∈*a*∘*x*+*b*∘*y*_ *β*(*z*)⊆(*α*(*a*) ∪ *β*(*x*)) ∪ (*α*(*b*) ∪ *β*(*y*)),
2. $\tilde{g}(x)\subseteq \tilde{f}(1)$ *and β*(*x*)⊇*α*(1),

for all *a*, *b* ∈ *F* and all *x*, *y* ∈ *V*.

**Proof**

Assume that $\langle (\tilde{g},\beta );V\rangle$ is a double-framed soft hypervector space of *V* related to $\langle (\tilde{f},\alpha );F\rangle$. The second condition follows from Proposition 11(2) and Definition 10(4). Let *a*, *b* ∈ *F* and *x*, *y* ∈ *V*. Then,

(20) ⋂z∈a∘x+b∘yg~(z) =⋂z∈u+vu∈a∘x,v∈b∘yg~(z)⊇(f~(a)∩g~(x))∩(f~(b)∩g~(y)),⋃z∈a∘x+b∘yβ(z) =⋃z∈u+vu∈a∘x,v∈b∘yβ(z)⊆(α(a)∪β(x))∪(α(b)∪β(y)).

Conversely suppose the conditions (1) and (2) are true. For all *x*, *y* ∈ *V*, we have

(21) g~(x+y)⊇⋂z∈1∘x+1∘yg~(z)⊇(f~(1)∩g~(x))∩(f~(1)∩g~(y))⊇g~(x)∩g~(y),β(x+y)⊆⋃z∈1∘x+1∘yβ(z)⊆(α(1)∪β(x))∪(α(1)∪β(y))⊆β(x)∪β(y).

Since $\langle (\tilde{f},\alpha );F\rangle$ is a double-framed soft subfield of *F*, we have $\tilde{g}(a)\subseteq \tilde{f}(1)\subseteq \tilde{f}(0)$, $\tilde{g}(a)\subseteq \tilde{f}(1)\subseteq \tilde{f}(-1)$, *β*(*a*)⊇*α*(1)⊇*α*(0), and *β*(*a*)⊇*α*(1)⊇*α*(−1). Note that 0 ∈ 0∘*x* for all *x* ∈ *V*. It follows that

(22) g~(−x)⊇⋂y∈0∘x+(−1)∘xg~(y)⊇(f~(0)∩g~(x))∩(f~(−1)∩g~(x))=g~(x)∩g~(x)=g~(x),

(23) β(−x)⊆⋃y∈0∘x+(−1)∘xβ(y)⊆(α(0)∪β(x))∪(α(−1)∪β(x))=β(x)∪β(x)=β(x),

for all *x* ∈ *V*. Let *a* ∈ *F* and *x* ∈ *V*. Then,

(24) ⋂y∈a∘xg~(y)⊇⋂y∈u+vu∈0∘x,v∈a∘xg~(y)⊇(f~(0)∩g~(x))∩(f~(a)∩g~(x))=g~(x)∩(f~(a)∩g~(x))=f~(a)∩g~(x),⋃y∈a∘xβ(y)⊆⋃y∈u+vu∈0∘x,v∈a∘xβ(y)⊆(α(0)∪β(x))∪(α(a)∪β(x))=β(x)∪(α(a)∪β(x))=α(a)∪β(x).

Clearly, $\tilde{f}(1)⊇\tilde{g}(\theta )$ and *α*(1)⊆*β*(*θ*). Therefore, $\langle (\tilde{g},\beta );V\rangle$ is a double-framed soft hypervector space of *V* related to $\langle (\tilde{f},\alpha );F\rangle$.

**Theorem 14**

Let *V* be a hypervector space over *F* and $\langle (\tilde{f},\alpha );F\rangle$ a double-framed soft subfield of *F*. If a DFS-set $\langle (\tilde{g},\beta );V\rangle$ over *V* is a double-framed soft hypervector space of *V* related to $\langle (\tilde{f},\alpha );F\rangle$, then the nonempty *γ*-inclusive set,

(25) $$\begin{array}{c} i_{V}\left(\tilde{g};\gamma \right)≔\left\{x\in V\;|\;\gamma \subseteq \tilde{g}\left(x\right)\right\}, \end{array}$$

and the nonempty *δ*-exclusive set,

(26) $$\begin{array}{c} e_{V}\left(\beta ;\delta \right)≔\left\{x\in V\;|\;\delta ⊇\beta \left(x\right)\right\}, \end{array}$$

of $\langle (\tilde{g},\beta );V\rangle$ are subhypervector spaces of *V* over the fields $i_{F}(\tilde{f};\gamma )$ and *e*_*F*_(*α*; *δ*), respectively, for all *γ*, *δ* ∈ *P*(*U*).

**Proof**

Let $x,y\in i_{V}(\tilde{g};\gamma )$. Then, $\tilde{g}(x)⊇\gamma$ and $\tilde{g}(y)⊇\gamma$. It follows that

(27) g~(x−y)=g~(x+(−y))⊇g~(x)∩g~(−y)⊇g~(x)∩g~(y)⊇γ.

Hence, $x-y\in i_{V}(\tilde{g};\gamma )$. Note that $i_{F}(\tilde{f};\gamma )$ is a subfield of *F*. Let $a\in i_{F}(\tilde{f};\gamma )$, $x\in i_{V}(\tilde{g};\gamma )$, and *y* ∈ *a*∘*x*. Then,

(28) $$\begin{array}{c} \tilde{g}\left(y\right)⊇\underset{z\in a\circ x}{⋂}\tilde{g}\left(z\right)⊇\tilde{f}\left(a\right)\cap \tilde{g}\left(x\right)⊇\gamma , \end{array}$$

and so $y\in i_{V}(\tilde{g};\gamma )$ which shows that $a\circ x\subseteq i_{V}(\tilde{g};\gamma )$. Therefore, $i_{V}\left(\tilde{g};\gamma \right)$ is a hypervector space over the field $i_{F}(\tilde{f};\gamma )$ for all *γ* ∈ *P*(*U*). Let *x*, *y* ∈ *e*_*V*_(*β*; *δ*). Then, *β*(*x*)⊆*δ* and *β*(*y*)⊆*δ*. It follows that

(29) β(x−y)=β(x+(−y))⊆β(x)∪β(−y)⊆β(x)∪β(y)⊆δ.

Hence, *x* − *y* ∈ *i*_*V*_(*β*; *δ*). Note that *e*_*F*_(*α*; *δ*) is a subfield of *F*. Let *a* ∈ *e*_*F*_(*α*; *δ*), *x* ∈ *i*_*V*_(*β*; *δ*), and *y* ∈ *a*∘*x*. Then,

(30) $$\begin{array}{c} \beta \left(y\right)\subseteq \underset{z\in a\circ x}{⋃}\beta \left(z\right)\subseteq \alpha \left(a\right)\cup \beta \left(x\right)\subseteq \delta , \end{array}$$

and so *y* ∈ *i*_*V*_(*β*; *δ*) which shows that *a*∘*x*⊆*i*_*V*_(*β*; *δ*). Therefore, *i*_*V*_(*β*; *δ*) is a hypervector space over the field *e*_*F*_(*α*; *δ*) for all *δ* ∈ *P*(*U*).

**Corollary 15**

Let *V* be a hypervector space over *F* and $\langle (\tilde{f},\alpha );F\rangle$ a double-framed soft subfield of *F*. If a DFS-set $\langle (\tilde{g},\beta );V\rangle$ over *V* is a double-framed soft hypervector space of *V* related to $\langle (\tilde{f},\alpha );F\rangle$, then the nonempty double-framed soft including set

(31) $$\begin{array}{c} DF_{V}{\left(\tilde{g},\beta \right)}_{\left(\gamma ,\delta \right)}≔\left\{x\in V\;|\;\gamma \subseteq \tilde{g}\left(x\right),\;\delta ⊇\beta \left(x\right)\right\}, \end{array}$$

of $\langle (\tilde{g},\beta );V\rangle$, is a subhypervector space of *V* over the field $DF_{F}{\left(\tilde{f},\alpha \right)}_{\left(\gamma ,\delta \right)}$ for all *γ*, *δ* ∈ *P*(*U*).
